# 吉瑞替尼一线联合化疗治疗FLT3突变阳性初诊急性髓系白血病16例的疗效及安全性分析

**DOI:** 10.3760/cma.j.cn121090-20240615-00224

**Published:** 2024-12

**Authors:** 雨田 雷, 晓丽 赵, 鸣 洪, 文洁 刘, 倩 孙, 思轩 钱, 帅 王, 雨 朱

**Affiliations:** 南京医科大学第一附属医院，江苏省人民医院血液科，南京 210029 Department of Hematology, Jiangsu Province Hospital, the First Affiliated Hospital of Nanjing Medical University, Nanjing 210029, China

## Abstract

为评估吉瑞替尼联合化疗治疗初诊FLT3突变阳性急性髓系白血病（AML）的疗效与安全性，我们回顾性收集于江苏省人民医院就诊的16例初诊FLT3突变阳性AML患者的临床资料。患者接受经典“3+7”方案或VA（维奈克拉+阿扎胞苷）方案诱导治疗，并均在检出FLT3-ITD/TKD突变后加用吉瑞替尼。16例患者中，男12例，女4例，中位年龄为52.5（15～76）岁；FLT3-ITD突变15例，FLT3-TKD突变1例。经过1个周期治疗后，完全缓解（CR）/CR伴不完全血细胞恢复（CRi）率达到93.8％（15/16），其中13例患者达到流式细胞术检测的可检测残留病（MRD）阴性。巩固治疗期间所有患者均达到CR_MRD−_，14例进行二代测序检测并均达FLT3突变阴性。截至2024年5月，中位随访时间为18个月，12个月总生存率和无复发生存率均为73.9％。诱导期间9例（56.2％）患者出现感染性发热，巩固与维持治疗期间3例患者发生3级QTc延长，治疗相关不良反应总体可耐受。

## Abstract (en)

This study aimed to assess the efficacy and safety of gilteritinib combined with chemotherapy in treating newly diagnosed FLT3-mutated acute myeloid leukemia（AML）. We retrospectively collected clinical data from 16 patients newly diagnosed with FLT3-mutated AML at Jiangsu Province Hospital. Patients received induction therapy with the classic “3 + 7” regimen or the VA regimen, and all patients were immediately supplied with gilteritinib after detecting FLT3-ITD/TKD mutations. Of the 16 patients, 12 were male and 4 were female, with a median age of 52.5 years（range: 15–76 years）. Additionally, 15 patients had FLT3-ITD mutations and 1 had FLT3-TKD mutation. The complete remission（CR/CRi）rate was 93.8％（15/16）after the first cycle of gilteritinib-based induction therapy, with 13 patients achieving MRD negativity detected with flow cytometry. All patients achieved a CR_MRD−_ during the consolidation phase. FLT3 mutation clearance was achieved among all 14 patients who underwent next-generation sequencing（NGS）analysis. The 12-month overall survival and relapse-free survival rates were both 73.9％, respectively, with a median followup of 18 months. Nine（56.2％）patients experienced infectious fever during the induction therapy. Three patients had grade 3 QTc prolongation during consolidation and maintenance therapy. Treatment-related adverse events were generally tolerable.

急性髓系白血病（AML）中FMS样酪氨酸激酶3（FLT3）突变的发生率约为30％，其中两个最主要的类型是内部串联重复（ITD）和酪氨酸激酶结构域突变（TKD）[1]–[2]。伴有FLT3-ITD突变的患者被认为预后不良，表现为较FLT3野生型更低的完全缓解（CR）率和更短的生存期[3]–[5]，FLT3抑制剂的应用有望改善这类患者的不良预后。奎扎替尼、吉瑞替尼、克拉尼布等第二代FLT3抑制剂靶向FLT3的特异性较强，有更高的亲和力，脱靶效应较第一代抑制剂显著减弱。目前我国可及的FLT3抑制剂包括索拉非尼和吉瑞替尼，而吉瑞替尼是其中唯一的第二代Ⅰ型FLT3抑制剂，对FLT3-ITD和TKD突变均有效[6]，多项临床研究显示其单药或联合化疗对初诊和复发/难治AML均有良好疗效[7]–[11]。本中心将吉瑞替尼与常规化疗联合治疗初诊的FLT3突变阳性AML，并将其疗效和安全性进行总结。

## 病例与方法

1. 病例：纳入2021年11月至2023年8月间南京医科大学第一附属医院收治的16例接受一线吉瑞替尼联合化疗的初诊AML伴FLT3突变患者。回顾性收集并分析这组患者的基线特征、疗效、安全性数据。所有患者在初诊时均接受骨髓细胞形态学、流式细胞术免疫分型、G显带法染色体核型分析、二代测序（NGS）基因突变检测，并应用多重巢式逆转录PCR或全转录组测序筛查融合基因。所有患者的诊断参照第五版WHO诊断标准[12]。依据2022年欧洲白血病网（ELN2022）成人AML诊断和治疗建议对患者进行预后危险度分层[13]。本研究经江苏省人民医院伦理委员会批准（2023-SR-284），所有患者均签署知情同意书。

2. FLT3突变检测：采用PCR法对FLT3-ITD进行检测，上游引物：5′-CAATTTAGGTATGAAAGCCAGCTACAGAT-3′；下游引物：5′-CTTTCAGCATTTTGACGGCAACCT-3′。等位基因比例（allelic ratio, AR）用于计算FLT3-ITD突变比例，AR＝FLT3-ITD突变型/野生型。等位基因突变频率（VAF）用于计算FLT3-TKD突变比例。

3. 治疗方案：诊断后根据患者体能状况及主管医师评估耐受情况选择标准IA方案（去甲氧柔红霉素12 mg/m^2^第1～3天，阿糖胞苷100 mg/m^2^第1～7天）或VA方案（阿扎胞苷100 mg/m^2^第1～7天，维奈克拉100 mg第1天，200 mg第2天，300 mg第3天，400 mg第4～28天）诱导治疗，在检出FLT3-ITD/TKD突变后立即加用吉瑞替尼80 mg每日1次治疗，全部患者启动吉瑞替尼时间在开始化疗7 d以内。1个疗程未获得CR者更换化疗方案再次诱导。根据患者意愿，部分体能状况良好者在第1次CR（CR_1_）后进行异基因造血干细胞移植（allo-HSCT），其他患者根据体能状态接受巩固治疗，方案包括：体能良好者使用中剂量阿糖胞苷3～4个周期，体能欠佳者使用VA方案6个周期。巩固治疗全疗程仍持续吉瑞替尼80 mg每日1次联合治疗，并在巩固治疗结束后继续吉瑞替尼80 mg每日1次单药维持治疗至少2年。

4. 疗效评价标准：每周期治疗后进行骨髓细胞形态学、多参数流式细胞术检查以评估疗效，疗效评估标准依据ELN2022指南[13]，分为CR、可检测残留病（MRD）阴性的CR（CR_MRD−_）、CR伴血细胞不完全恢复（CRi）、未缓解（NR）。MRD阴性指低于流式细胞术检测下限。

5. 随访：末次随访日期为2024年5月1日，中位随访时间为586（285～908）d。总生存（OS）期是从治疗开始到死亡的时间；无复发生存（RFS）期是从获得CR至复发或因任何原因死亡的时间。

6. 统计学处理：采用Kaplan-Meier法进行生存分析计算生存率。GraphPad Prism 9软件进行统计学分析和绘图。

## 结果

1. 临床特征：16例患者中男12例，女4例，中位年龄为52.5（15～76）岁；FLT3-ITD突变15例，FLT3-TKD突变1例。根据FAB分型，M_1_型6例，M_2_型5例，M_4_型2例，M_5_型3例。12例（75.0％）合并NPM1突变，6例（37.5％）合并DNMT3A突变，4例（25.0％）合并IDH2突变，2例（12.5％）合并KRAS突变。共4例在初诊时检出融合基因，分别为DEK::NUP214、RUNX1∷RUNX1T1、DDX10∷SKA3和CBFB∷MYH11融合。根据ELN2022危险度分层，预后良好组3例，预后中等组12例，预后不良组1例。初诊时患者的临床特征具体见表1。

**表1:** 16例接受吉瑞替尼联合化疗治疗的FLT3突变急性髓系白血病患者的临床特征

例号	性别	年龄（岁）	FAB分型	染色体核型	FLT3突变类型	其他合并突变	融合基因	2022ELN危险度分层
1	女	61	M_1_	46,XX[20]	ITD	NPM1、DNMT3A、CUX1	阴性	中危
2	女	33	M_2_	46,XX[6]	ITD	NRAS	DEK∷NUP214	高危
3	男	30	M_2_	45,X,-Y,t（8;21）（q22;q22）[10]	ITD	无	RUNX1∷RUNX1T1	低危
4	女	53	M_1_	46,XX[12]	ITD	NPM1、TET2	阴性	中危
5	女	58	M_1_	未见分裂象	ITD	DNMT3A、IDH2、NPM1	阴性	中危
6	女	76	M_4_	46,XX[20]	ITD	DNMT3A、KRAS、NPM1	DDX10∷SKA3	中危
7	女	60	M_1_	46,XX[20]	ITD	NPM1	阴性	中危
8	女	60	M_2_	46,XX[20]	ITD	DNMT3A、CEBPA、CSF3R	阴性	中危
9	女	15	M_1_	46,XX[20]	TKD	NPM1、GATA2、KMT2A	阴性	低危
10	男	50	M_1_	46,XY[20]	ITD	IDH2、NPM1	阴性	中危
11	女	46	M_5_	46,XX[11]	ITD	DNMT3A、NPM1、DDX41	阴性	中危
12	女	35	M_5_	46,XX[20]	ITD	NPM1、BCOR	阴性	中危
13	女	55	M_2_	46,XX[20]	ITD	IDH2、NPM1	阴性	中危
14	男	52	M_2_	46,XY[20]	ITD	IDH2、NPM1	阴性	中危
15	女	59	M_5_	46,XX[5]	ITD	NPM1、DNMT3A、WT1	阴性	中危
16	男	36	M_4_	47,XY,inv（16）（p13q22）,+22[8]/46,XY[2]	ITD	KRAS、EPPK1、MBD2	CBFB∷MYH11	低危

**注** ELN：欧洲白血病网

2. 疗效分析：16例患者中，3例接受吉瑞替尼联合标准IA方案诱导治疗，13例接受吉瑞替尼联合VA方案诱导治疗。1个周期吉瑞替尼联合诱导治疗后CR/CRi率为93.8％（15/16），其中13例获得CR_MRD−_，1例形态学CR和1例CRi。吉瑞替尼联合VA方案组的CR/CRi率为92.3％（12/13），联合IA方案组3例均达CR/CRi。未达CR的患者在第1周期吉瑞替尼联合VA方案诱导治疗失败后，更换吉瑞替尼联合IA方案再诱导治疗后达CR。巩固治疗期间所有患者均获得CR_MRD−_，14例（87.5％）患者均获得以NGS法检测的FLT3突变阴性，第1周期治疗后患者的FLT3-ITD AR或TKD VAF见表2。

**表2:** 吉瑞替尼联合化疗诱导治疗第1周期前后FLT3-ITD AR或TKD VAF变化

例号	治疗前FLT3-ITD AR或TKD VAF	1个周期诱导后FLT3-ITD AR或TKD VAF
1	7.673	未检测
2	0.450	0.090
3	1.167	0.436
4	0.711	0.022
5	0.230	未检测
6	0.480	未检测
7	1.676	0.022
8	0.191	0
9	34.091	0
10	0.543	0.014
11	0.165	0.023
12	0.450	0
13	0.119	0
14	0.202	0
15	4.338	0
16	0.047	0

**注** AR：等位基因比例；VAF：等位基因突变频率

3例（18.8％）患者在CR_1_接受allo-HSCT，缓解至进行移植的中位时间为3.0（2.7～3.3）个月，移植造血重建后继续以吉瑞替尼维持治疗，中位随访24（21～27）个月时无患者移植后复发。4例（25.0％）接受5个周期吉瑞替尼联合VA方案作为巩固治疗，后吉瑞替尼单药维持治疗，其中1例在缓解13个月后形态学复发。6例（37.5％）至少接受过1个周期以中剂量阿糖胞苷为基础的巩固治疗，所有患者均已进入维持治疗阶段，目前无复发。仅有1例（6.3％）患者目前仍处于吉瑞替尼联合VA方案巩固治疗中。2例患者在获得CR/CRi后自行停止治疗，分别于出院3个月和2个月后因疾病进展死亡。

截至末次随访，所有患者中共3例复发，其中1例发生于维持治疗阶段，2例发生于自行停止治疗后，12个月OS率和RFS率均为73.9％（标准误11.3％），无复发生存率为62.5％（图1）。中位OS期与RFS期均未达到。

**图1:**
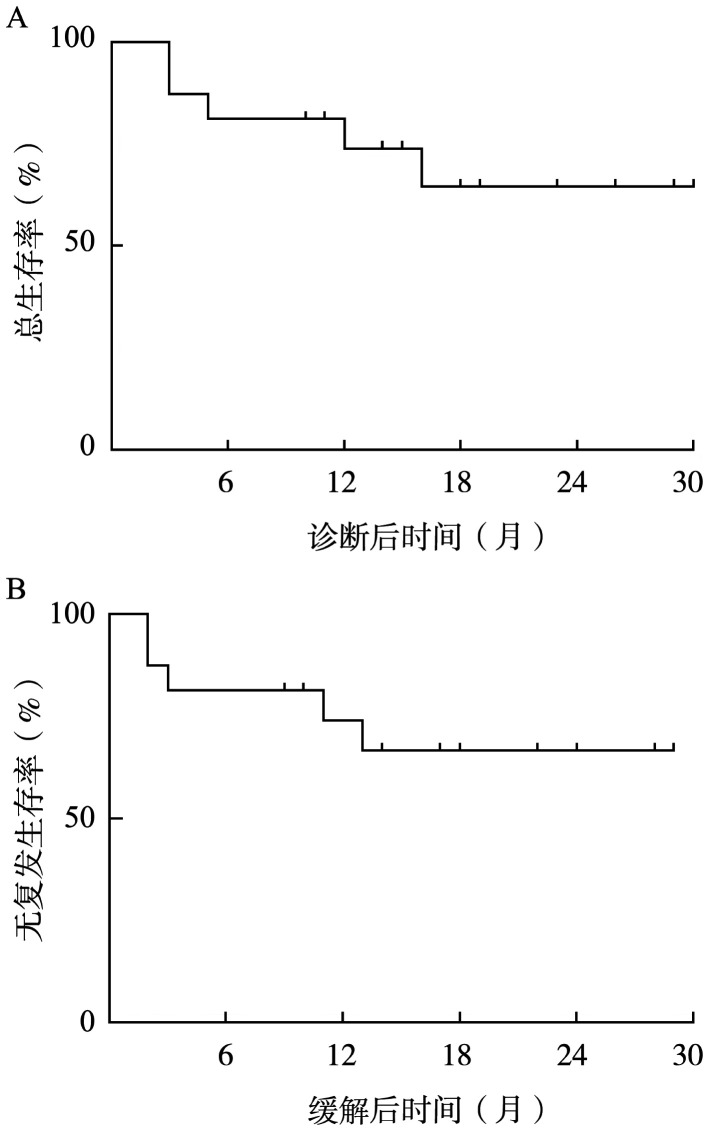
16例FLT3突变阳性急性髓系白血病患者的总生存曲线（A）和无复发生存曲线（B）

3. 安全性分析：诱导治疗期间所有患者均发生4级血液学不良反应。在第1周期诱导治疗后获得缓解的患者中，VA方案联合吉瑞替尼治疗的患者脱离中性粒细胞缺乏（ANC>0.5×10^9^/L）中位时间为28（15～51）d，血小板恢复（PLT>50×10^9^/L）中位时间为26（10～68）d；IA方案联合吉瑞替尼治疗者分别为20（17～23）d和20（20～23）d。

诱导期间所有患者均观察到食欲减退和疲劳，此外发生率较高的不良反应还有便秘（87.5％）、转氨酶升高（75％）、低钾血症（75％），但症状较轻微，经对症支持治疗后均好转。诱导及巩固治疗过程中发生的3～4级非血液学不良反应见表3。诱导治疗期间，吉瑞替尼联合VA方案和IA方案最常见的3～4级非血液学不良反应均为感染性发热，发生率分别为53.8％（7/13）和66.7％（2/3），无肿瘤溶解综合征或其他严重脏器不良反应发生，无早期死亡病例。巩固治疗期间，75.0％（6/8）接受吉瑞替尼联合强化疗的患者发生3～4级感染性发热，并有37.5％（3/8）发生败血症；联合VA方案治疗的患者仅20.0％（2/10）发生3～4级感染性发热。吉瑞替尼联合VA方案治疗的患者中30.0％（3/10）发生3级QTc延长，未导致严重心律失常事件，吉瑞替尼减量或暂停后均好转，并且所有患者均能够耐受重启吉瑞替尼80 mg每日1次治疗；未在吉瑞替尼联合强化疗作为巩固治疗的患者中观察到3～4级QTc延长。

**表3:** 吉瑞替尼联合化疗诱导及巩固治疗过程中发生的3～4级不良反应［例（％）］

不良反应	诱导治疗阶段		巩固治疗阶段	
	联合VA方案（13例）	联合IA方案（3例）	联合VA方案（10例）	联合中剂量Ara-C（8例）
感染性发热	7（53.8）	2（66.7）	2（20.0）	6（75.0）
败血症	0（0）	1（33.3）	0（0）	3（37.5）
QTc间期延长	0（0）	0（0）	3（30.0）	0（0）
低钠血症	1（7.7）	0（0）	0（0）	1（12.5）
低钾血症	1（7.7）	1（33.3）	1（10.0）	2（25.0）
肺栓塞	1（7.7）	0（0）	0（0）	0（0）

**注** VA：阿扎胞苷+维奈克拉；IA：去甲氧柔红霉素+阿糖胞苷；Ara-C：阿糖胞苷

## 讨论

FLT3突变通过多种通路广泛影响细胞增殖、分化和存活，与预后不良相关[14]。在传统化疗时代，FLT3-ITD对化疗敏感性差，在标准化疗下缓解率低，即使早期移植也无法改善其不良预后[15]。一项回顾性研究显示，47例接受allo-HSCT的患者复发率高达45％，中位OS期仅17个月[5]。但分子靶向治疗药物的问世与迭代带来患者缓解率的提高与生存期的延长，在ELN2022标准下所有伴FLT3-ITD突变的AML均被归为中危组[13]。

吉瑞替尼是唯一在我国获批的二代FLT3抑制剂，相较于索拉非尼、米哚妥林等一代FLT3抑制剂，吉瑞替尼有着特异性高、脱靶效应少的特点[16]，对FLT3-ITD和TKD突变都有抑制作用[6],[17]。Ⅲ期 ADMIRAL研究纳入371例≥18岁的FLT3突变复发/难治AML患者，吉瑞替尼组CR/CR伴部分血液学恢复率（34.0％对15.3％）和复合完全缓解率（54.3％ 对21.8％）显著高于接受挽救性化疗的患者；吉瑞替尼治疗的患者OS期显著延长（中位OS期：9.3个月对5.6个月），1年OS率分别为37.1％和16.7％[7]。鉴于在复发难治人群中的疗效，近年来吉瑞替尼的使用也逐渐被前移到一线治疗中，以期提高一线诱导治疗成功率和减少缓解后复发。Pratz等[10]报道，36例新诊断的FLT3突变阳性AML患者接受吉瑞替尼联合IA或DA方案诱导，1个疗程CR/CRi率为89％，中位生存期为46.1个月，且耐受性良好。Short等[11]将吉瑞替尼与维奈克拉、阿扎胞苷联合用于30例中位年龄为71岁的初诊FLT3突变阳性AML患者的诱导治疗，CR/CRi率高达96％，18个月的RFS和OS率分别为71％和72％。这些研究证实吉瑞替尼与传统的细胞毒药物标准化疗或维奈克拉为基础的分子靶向治疗方案联用在一线治疗中有极高的CR率，生存数据良好。目前国内对于吉瑞替尼疗效的报道大多为个案，且主要应用于复发/难治阶段。本研究中的16例患者均为一线治疗，且13例（81.3％）接受VA方案联合吉瑞替尼诱导，未使用细胞毒药物，治疗过程经历的不良反应级别低，安全性很高。吉瑞替尼联合VA方案未达CR的1例患者伴RUNX1∷RUNX1T1融合基因，考虑与该亚组的人群对维奈克拉不敏感有关。其他12例吉瑞替尼联合VA方案诱导治疗的患者均达到缓解，表明吉瑞替尼联合VA方案的疗效与联合“3+7方案”等传统细胞毒药物的疗效相当，而安全性明显优于传统治疗。

在巩固与维持治疗阶段，我们观察到持续使用吉瑞替尼后获得了很高的累积FLT3突变清除率，14例在巩固治疗期间均获得以NGS方法检测的FLT3突变阴性。本研究中，仅有3例（18.8％）患者在CR_1_接受allo-HSCT，移植后造血重建后继续以吉瑞替尼维持治疗，移植患者在中位随访24（21～27）个月时无复发，获得了理想的RFS。同时未移植的13例患者有2例在获得CR/CRi后自行停止治疗，分别于出院3个月和2个月后因疾病进展死亡，1例在缓解13个月后形态学复发，其余10例患者中位随访18个月均未出现复发。值得注意的是，其中5例仅VA方案治疗，未用任何细胞毒药物，也获得了满意的RFS，提示在吉瑞替尼加入一线治疗的前提下，相当比例的患者可在CR_1_不行allo-HSCT也能获得理想的疗效。

本研究表明，吉瑞替尼联合化疗一线治疗FLT3突变阳性的AML具有极高的CR率，缓解程度深，即使在CR_1_未进行allo-HSCT的人群中也获得了良好的无复发生存。吉瑞替尼在一线治疗中诱导、巩固、维持阶段均表现出良好的耐受性，与常规化疗方案联合并未观察到不良反应增加。
